# IDH1 Mutation Induces HIF-1*α* and Confers Angiogenic Properties in Chondrosarcoma JJ012 Cells

**DOI:** 10.1155/2022/7729968

**Published:** 2022-02-14

**Authors:** Xiaoyu Hu, Luyuan Li, Josiane E. Eid, Chao Liu, Jinming Yu, Jinbo Yue, Jonathan C. Trent

**Affiliations:** ^1^Department of Oncology, Renmin Hospital of Wuhan University, Wuhan, China; ^2^Department of Radiation Oncology, Shandong Cancer Hospital and Institute, Shandong First Medical University and Shandong Academy of Medical Sciences, Jinan, Shandong, China; ^3^Department of Medicine, Division of Medical Oncology, University of Miami Miller School of Medicine, Miami, USA; ^4^Sylvester Comprehensive Cancer Center, University of Miami Miller School of Medicine, Miami, USA

## Abstract

Chondrosarcoma is a group of primary bone cancers that arise from transformed cells of chondrocytic lineage. Tumor recurrence and metastasis are devastating for patients with chondrosarcoma since there are no effective treatment options. IDH mutations occur in over 50% of tumors from patients with conventional or dedifferentiated chondrosarcomas and represent an attractive target for therapy. However, their role in the pathogenesis of chondrosarcoma remains largely unknown. In this study, we sought to determine the association of IDH mutation and HIF-1*α* in chondrosarcoma. We used the chondrosarcoma JJ012 cell line and its derived CRISPR/Cas9 mutant IDH1 (IDH1^mut^) knockout (KO) cells. RNA-Seq data analysis revealed downregulation of several HIF-1*α* target genes upon loss of IDH1^mut^. This was associated with reduced HIF-1*α* levels in the IDH1^mut^ KO cells and tumors. Loss of IDH1^mut^ also attenuated the expression of angiogenic markers in tumor tissues and abrogated the angiogenic capacity of JJ012 cells. Moreover, we observed that exogenous expression of HIF-1*α* significantly promoted anchorage-independent colony-formation by IDH1^mut^ KO cells. These results suggest IDH1 mutation confers angiogenic and tumorigenic properties of JJ012 cells by inducing HIF-1*α*. Thus, the HIF pathway represents a promising candidate for combinatorial regimens to target IDH1 mutated chondrosarcomas.

## 1. Introduction

Chondrosarcomas constitute a heterogeneous group of primary bone cancers characterized by the formation of a hyaline cartilaginous matrix. Following osteosarcoma, chondrosarcoma is the second most common bone malignancy, accounting for 20% to 27% of primary bone tumors [1, 2]. Approximately 85% of chondrosarcomas are the conventional subtypes which can be further classified into central, peripheral, and periosteal lesions. The remaining 10–15% consist of rare subtypes including dedifferentiated, mesenchymal, clear cell, and myxoid chondrosarcoma. Chondrosarcomas are notoriously resistant to chemotherapy and radiotherapy, and surgery is the backbone treatment for most localized tumors [2, 3]. Chondrosarcomas tend to recur with more aggressive behavior than the original neoplasm following initial tumor resection. As a result, many patients develop metastatic disease which is nearly uniformly fatal. Due to lack of effective treatment strategies for recurrent or metastatic chondrosarcoma, high-grade conventional and dedifferentiated chondrosarcomas have poor prognosis [1, 4]. Current studies focus on clarifying the link between molecular events and pathogenesis of this malignancy and developing new molecularly targeted therapies for advanced diseases.

Isocitrate dehydrogenase (IDH) mutation is among one of the promising therapeutic targets. IDH1/2 mutations were found in 71% of conventional chondrosarcomas and 57% of dedifferentiated chondrosarcomas [4, 5], as well as in gliomas and acute myeloid leukemia [6, 7], suggesting a potential role for aberrant IDH function in the pathogenesis of these malignancies. IDHs normally convert isocitrate to *α*-ketoglutarate (*α*-KG). However, mutant IDHs lose the ability to catalyze this reaction but instead gain a neomorphic function of reducing *α*-KG to D-2-hydroxyglutarate (D-2HG), which has been reported to accumulate at high levels in IDH1/2-mutated tumors [8, 9]. D-2HG and *α*-KG are structurally similar. Thus, accumulated D-2HG is thought to act as an oncometabolite through the inhibition of various *α*-KG-dependent enzymes including the TET family of 5-methycytosine hydroxylases, JumonjiC domain-containing histone demethylases (JHDMs), and the Prolyl Hydroxylase Domain-Containing Proteins (PHDs) [9–11].

HIF-1*α*, a key hypoxia-inducible transcription factor, is associated with tumor development as it functions as a master regulator of genes involved in angiogenesis, glucose metabolism, and other cellular pathways [12]. HIF-1*α* overexpression is correlated with disease progression, chemo-radio-resistance, and increased patient mortality in certain cancers [13–15]. The stability and transcriptional activity of HIF-1*α* are regulated by PHDs (PHD1, PHD2, and PHD3). Under normal oxygen conditions, PHDs utilize *α*-KG and O_2_ to hydroxylate a conserved proline in HIF-1*α*, leading to Von Hippel-Lindau- (VHL-) mediated ubiquitination and subsequent proteasomal degradation of HIF-1*α* [16, 17]. In hypoxic conditions, however, these hydroxylation events cannot proceed efficiently, resulting in accumulation of HIF-1*α* [18]. Interestingly, studies examining the effects of D-2HG on PHDs and HIF-1*α* in IDH-mutant gliomas and leukemias have yielded conflicting results. It has been reported that D-2HG competitively inhibits the activity of PHDs as mentioned above and thus leads to increased levels of HIF-1*α* [18, 19]. Conversely, D-2HG was shown to act as an activator rather than an inhibitor of PHDs, ultimately leading to decreased levels of HIF-1*α* [20, 21]. In contrast to the emerging knowledge in gliomas and leukemias, little is known regarding the effect of IDH mutation or D-2HG on HIF-1*α* activity in chondrosarcoma. Understanding their relationship would have great clinical importance in terms of developing novel targeted therapies for advanced chondrosarcomas.

In this study, we sought to determine the potential association of IDH mutation and HIF-1*α* in chondrosarcoma. We employed the IDH1-mutant chondrosarcoma JJ012 cell line, CRSPR/Cas9 mutant IDH1 (IDH1^mut^) knockout (KO) JJ012 clones, and their derived xenografts. We found that CRISPR/Cas9 knockout of IDH1^mut^ reduced HIF-1*α* levels *in vitro* and *in vivo*, leading to downregulation of HIF-1*α* target genes. Loss of IDH1^mut^ also decreased the expression of angiogenic markers in tumors and attenuated the angiogenic capacity of JJ012 cells. Moreover, we observed restoring HIF-1*α* levels with exogenous expression significantly enhanced the anchorage-independent growth of IDH1^mut^ KO cells. These results suggest IDH1 mutation confers tumorigenic and angiogenic properties by inducing HIF-1*α* in a JJ012 chondrosarcoma model.

## 2. Materials and Methods

### 2.1. Cell Culture

The human chondrosarcoma JJ012 and human chondrocyte C28 cell lines were kindly provided by Dr. Joel Block and Dr. Karina Galoian, respectively. JJ012 harbors a monoallelic IDH1 R132G mutation while C28 carries wildtype IDH1 (IDH1^wt^). JJ012 cells were cultured in RPMI-1640 medium (Lonza) supplemented with 10% fetal bovine serum (FBS) and 1% Penicillin/Streptomycin. C28 cells were grown in 1 : 1 DMEM/F12 medium (HyClone) supplemented with 10% FBS and 1% Penicillin/Streptomycin. Human umbilical vein endothelial cells (HUVECs) were obtained from Thermo Fisher Scientific and grown in Endothelial Cell Growth Medium 2 (EGM-2) (PromoCell) on 0.1% gelatin-coated plates. Cells were maintained at 37°C in a humidified air with 5% CO_2_.

### 2.2. IDH1 Knockout by CRISPR/Cas9 Technology

Knockout of IDH1^mut^ was achieved by the CRISPR/Cas9 system. The CRISPR/Cas9 plasmid products were purchased from Santa Cruz Biotechnology. Details of transfection, selection, and single-cell colonies propagation were previously described [22].

### 2.3. Measurement of D-2HG

Quantitative analyses of D-2HG were conducted by high-performance liquid chromatography–tandem mass spectrometry (HPLC-MS/MS) with single-reaction monitoring (SRM) scans. This was performed at MtoZ Biolabs (Boston, MA, USA).

### 2.4. Tumor Tissues

Tumor samples were obtained from mice-bearing chondrosarcoma xenografts which were derived from JJ012 parental and IDH1^mut^ KO cells, as previously described. All animal experiments were performed in compliance with the University of Miami Institutional Animal Care and Use Committee (IACUC)-approved protocol (No. 19-079).

### 2.5. RNA-Seq and Ingenuity Pathway Analysis (IPA)

RNA-Seq and IPA were described with details previously [22].

### 2.6. Quantitative Reverse Transcriptase Polymerase Chain Reaction (qRT-PCR)

Total RNA was extracted using the miRNeasy Mini Kit (Qiagen) according to the manufacturer's protocol. cDNA was synthesized from 2 *μ*g of total RNA using iScript a cDNA synthesis kit (Bio-Rad:1708891) in a 40 *μ*l total volume. qRT-PCR was set up with 20x TaqMan probes, 2 *μ*l of 1 : 5 diluted cDNA and TaqMan universal PCR Master Mix (Thermo Fisher Scientific) in 20 *μ*l total volume. Samples were run in triplicate on a Bio Rad CFX-96 real time PCR system. Gene expression levels were calculated using the 2^-*ΔΔ*Ct^ method [23]. Gene-specific TaqMan primers/probe sets include GAPDH (internal normalization control), VEGFA, VEGFC, EDN1, and SLC2A3.

### 2.7. Western Blotting

Cells were lysed in a Laemmli sample buffer (Bio-Rad) supplemented with 2-mercaptoethanol (Sigma-Aldrich). The lysates were centrifuged at 14,000 rpm for 10 min at 4° C. The supernatants were collected and denatured at 95°C for 10 min. Equal protein lysates were separated on 4-20% Mini-PROTEIN TGX precast gels (Bio-Rad) and transferred to nitrocellulose membranes (Pall Corporation). The following antibodies were used: anti-IDH1 (1 : 1000, Abcam, ab172964), anti-HIF-1*α* (0.5 *μ*g/ml, RD Systems, NB100-105), and anti-*β*-actin (1 : 5000, Cell Signaling).

### 2.8. Soft-Agar Colony Formation Assay

5 × 10^3^ cells were plated in a 0.3% top layer soft agar in RPMI-1640 with 10% FBS overlaid on a lower layer of a 0.5% basal agar. Cells were then maintained in regular incubator with 21% O_2_, or in a hypoxia chamber with 1% O_2_ at 37°C for 10-14 days. Colonies were stained with 2 mg/mL iodonitrotetrazolium chloride (Sigma-Aldrich), rinsed with PBS, and quantified with GelCount colony counter (Oxford Optronix).

### 2.9. Immunohistochemistry (IHC)

Immunohistochemical analysis was performed on 5-*μ*m sections cut from formalin-fixed, paraffin-embedded samples utilizing antibodies against CD31 (Servicebio, GB11063-2), HIF-1*α* (Bioworld, BS3514), VEGFA (Abcam, ab52917), and IDH1 (Abcam, ab172964) following a standard protocol. A semiquantitative evaluation method was applied as follows: the score obtained by the percentage of positive cells (0% = 0; 1–25% = 1, 26–50% = 2, 51–75% = 3, and >75% = 4) was multiplied by the score obtained by the staining intensity (no staining = 0, weak staining = 1, moderate staining = 2, and strong staining = 3). Scoring was evaluated by investigators who were blinded to the information of research subjects.

### 2.10. Vascular-Endothelial Tube Formation Assay

JJ012 parental and IDH1^mut^ KO cells were cultured with serum-supplemented RPMI-1640 overnight, followed by a 24 h starvation in serum-free medium. Secretome derived from the culture supernatant was then collected and centrifuged at 1200 rpm for 5 min to remove debris. The vascular-endothelial tube formation assay was performed with HUVEC cells in 24-well plates pre-coated with growth factor-reduced basal membrane Geltrex matrix (Thermo Fisher Scientific) and left to solidify for 30 min at 37°C. HUVEC cells were harvested with Trypsin/EDTA solution and the cell concentrations were determined in non-supplemented EGM-2. 2 × 10^5^ cells were seeded in each well and incubated with 1 ml of cell supernatant secretome for 24 hr. After incubation, cells were stained with Calcein AM (2 *μ*g/ml) for 30 min, rinsed with 1 mM CaCl_2_ and 0.5 mM MgCl_2_-supplemented PBS, and fixed with 4% paraformaldehyde in PBS for 25 min at room temperature. Capillary tubes were visualized with fluorescence microscopy. The total vascular-endothelial tube lengths were measured from randomly selected image fields per sample per group using NIH/Image J. At least five fields per well were examined, and each experimental condition was tested in triplicate. All HUVEC cells used in experiments underwent fewer than eight passages after resuscitation.

### 2.11. Statistical Analysis

GraphPad Prism 8 software was used for statistical analyses. *p* values were determined by unpaired, two-tailed *t* tests. *p* < 0.05 was considered statistically significant.

## 3. Results

### 3.1. Loss of IDH1^mut^ Leads to Downregulation of HIF-1*α* Target Genes in JJ012 Cells

**Figure 1:**
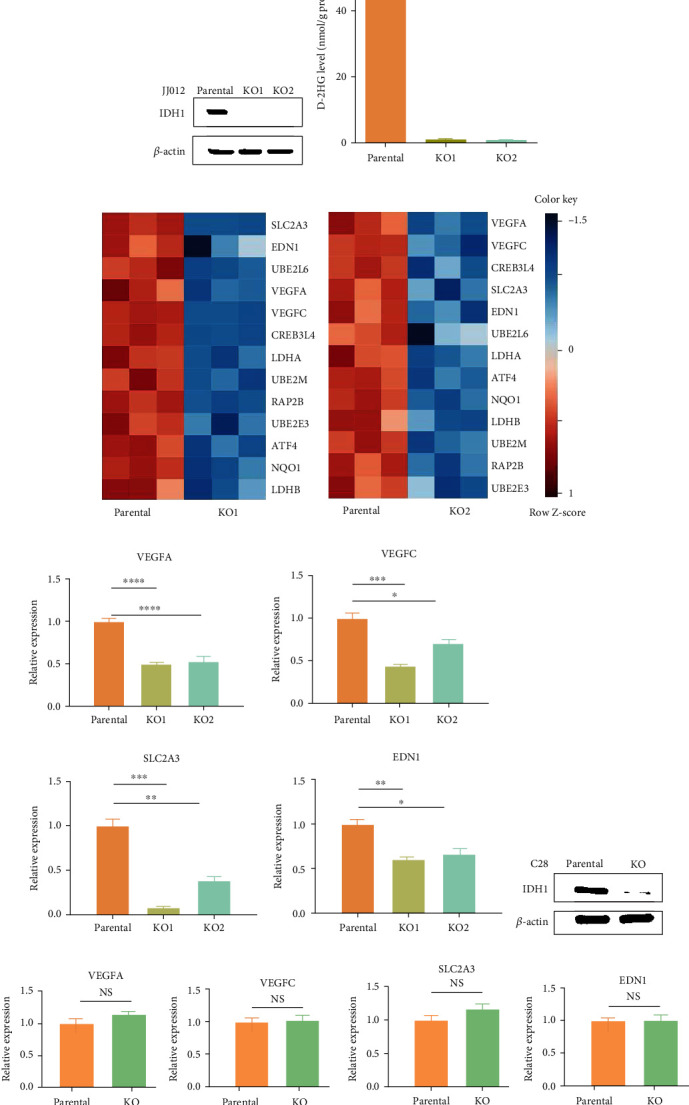
Downregulation of HIF-1*α* target genes upon loss of IDH1^mut^ in JJ012 cells. (a) Immunoblot shows depletion of IDH1 protein in two IDH1^mut^ KO clones of JJ012 cells. (b) HPLC-MS analysis indicates D-2HG production was nearly depleted in two IDH1^mut^ KO clones of JJ012 cells. (c) Heatmaps of HIF-1*α* target genes that are downregulated in both IDH1^mut^ KO clones compared to parental control of JJ012 cells. (d) mRNA expression of VEGFA, VEGFC, EDN1, and SLC2A3 in JJ012 cells was quantified by qRT-PCR. The amount of transcript was normalized to GAPDH, and the results are shown as fold-change relative to the parental control. Data are shown as mean ± SEM of triplicate values and are representative of three independent experiments. ∗*p* < 0.05, ∗∗*p* < 0.01, ∗∗∗*p* < 0.001. (e) Immunoblot shows levels of wildtype IDH1 in CRISPR/Cas9 KO C28 chondrocytes. (f) Bar graphs compare levels of VEGFA, VEGFC, EDN1, and SLC2A3 mRNA expression in parental and wildtype IDH1 KO C28 cells. NS: nonsignificant (*p* > 0.05).

In this study, we utilized the human chondrosarcoma JJ012 cell line which harbors an endogenous IDH mutation, and its derived two CRISPR/Cas9 IDH1^mut^ KO clones. Depletion of the IDH1 protein and reduced D-2HG levels in the two IDH1^mut^ KO clones are shown in Figures 1(a) and 1(b), respectively. To begin to understand the role of IDH mutation in chondrosarcoma tumorigenesis, we initially conducted an RNA-Seq analysis of JJ012 parental cells and its two IDH1^mut^ KO clones. The RNA-Seq analysis revealed an association between IDH mutation and aberrant activation of integrin signaling in chondrosarcoma [22]. Interestingly, in addition to mediators of cell adhesion and integrin-related pathways, we found in the transcriptome of JJ012 IDH1^mut^ KO cells many genes known to be involved in vasculogenesis. The downregulated HIF-1*α* target genes detected in both IDH1^mut^ KO clones are shown in Figure 1(c). These genes are implicated in glucose metabolism (SLC2A3, LDHA and LDHB) and angiogenesis (VEGFA and VEGFC). Downregulation of several well-established HIF-1*α* target genes including VEGFA, VEGFC, EDN1 and SLC2A3 in the IDH1^mut^ KO cells was further confirmed by qRT-PCR (Figure 1(d)). These results indicate that IDH mutation is associated with activation of the HIF-1*α* signaling pathway.

To be noted, both IDH1^wt^ and IDH1^mut^ were knocked out in our cell model. To confirm that it is the loss of IDH1^mut^ allele rather than that of IDH1^wt^ is responsible for the downregulation of the HIF-1*α* target genes, we utilized C28 cells, an immortalized human chondrocyte cell line that expresses IDH1^wt^ only [24]. A pool of C28 IDH1^wt^ KO cells with markedly reduced IDH1 levels was created using the CRISPR/Cas9 technique (Figure 1(e)). qRT-PCR analysis revealed comparable expression of the aforementioned HIF-1*α* target genes between C28 parental and IDH1^wt^ KO cells (Figure 1(f)), suggesting that loss of the IDH1^mut^ rather than of the IDH1^wt^ allele caused the downregulation of the HIF-1*α* target genes in JJ012 cells.

### 3.2. Loss of IDH1^mut^ Reduces HIF-1*α* Levels in JJ012 Cells and Tumor Tissues

**Figure 2:**
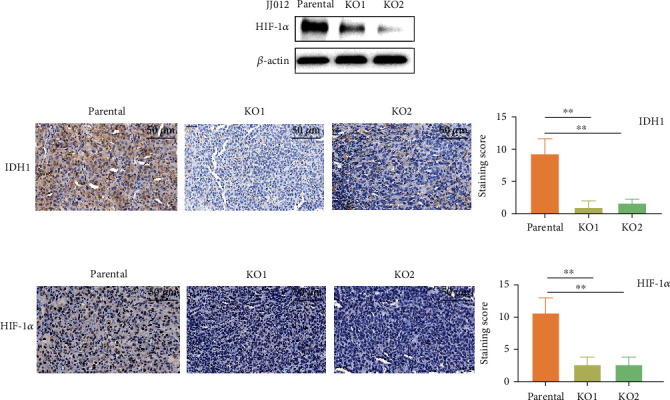
Loss of IDH1^mut^ suppresses HIF-1*α* in JJ012 cells and tumor tissues. (a) Immunoblots show HIF-1*α* expression in JJ012 parental and IDH1^mut^ KO cells; immunohistochemical (IHC) images show cytoplasmic IDH1 (b) and nuclear HIF-1*α* (c) expression in parental and IDH1^mut^ KO JJ012-derived xenografts. IHC staining was quantified with intensity scores as detailed in Materials and Methods. Scale bars are 50 *μ*m. Data represent mean ± SEM of values from four random fields. ∗∗*p* < 0.01.

HIF-1*α* protein levels are regulated by PHDs which destabilize the angiogenic transcription factor by post-translational proline hydroxylation under normoxic conditions. It has been reported that D-2HG competitively inhibits PHDs due to its structural similarity to *α*-KG, thereby causing accumulation of HIF-1*α* in IDH1-mutant glioma cells [19]. In our model, D-2HG production was almost completely suppressed in JJ012 IDH1^mut^ KO clones [22]. Therefore, it is rational to inquire whether the downregulation of HIF-1*α* target genes in the IDH1^mut^ KO cells is attributed to HIF-1*α* inhibition as a result of IDH1^mut^ loss and reduced D-2HG production. Indeed, HIF-1*α* expression appeared to be significantly decreased in the two IDH1^mut^ KO JJ012 cell lines compared to their parental control under normoxic conditions (Figure 2(a)). We then proceeded to ask whether HIF-1*α* was similarly regulated *in vivo*. For this we measured IDH1 expression in the chondrosarcoma xenografts derived from parental and IDH1^mut^ KO JJ012 cells that we have previously established (22). IHC analysis revealed significant reduction of IDH1 in the IDH1^mut^ KO tumors (Figure 2(b)). Importantly, HIF-1*α* levels were concomitantly reduced in the same tumors, thus confirming our *in vitro* findings. The staining score showed that HIF-1*α* expression was reduced by 70%-80% in the IDH1^mut^ KO tumors compared to parental controls (Figure 2(c)). These results demonstrate that knockout of IDH1^mut^ downregulates HIF-1*α in vitro* and *in vivo*, thus supporting the concept that IDH1 mutation promotes HIF-1*α* stabilization and its downstream signaling in our JJ012 chondrosarcoma model.

### 3.3. IDH1 Mutation Confers Angiogenic Properties in JJ012 Chondrosarcoma Cells

**Figure 3:**
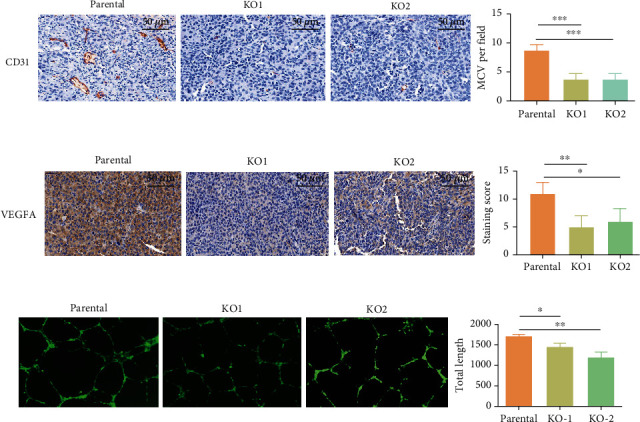
IDH1 mutation promotes angiogenic property of JJ012 cells. (a) IHC images show CD31 expression in JJ012 parental and IDH1^mut^ KO cell-derived xenografts. Right graph depicts number of CD31-positive microvessels (MCV) per field in each group. (b) IHC images show VEGFA expression in JJ012 parental and IDH1^mut^ KO cell-derived xenografts. VEGFA staining was quantified with intensity scores (right graph) as detailed in Materials and Methods. Scale bars are 50 *μ*m. Data represent mean ± SEM of values from at least four random fields. (c) Representative images and quantification (right graph) of endothelial tube formation. HUVECs were cultured in secretome-derived media from JJ012 parental and IDH1^mut^ KO cells. The total capillary tube lengths were measured from 9 to 12 randomly selected image fields per sample per group using NIH/Image J. ∗*p* < 0.05, ∗∗*p* < 0.01, ∗∗∗*p* < 0.001.

Angiogenesis represents an essential step in tumor proliferation, expansion, and metastasis, thus contributing to the pathology of virtually all human cancers. HIF-1*α* is a subunit of HIF-1, an oxygen-dependent transcriptional activator, which plays crucial roles in tumor angiogenesis and mammalian development [12]. HIF-1 activates transcription of genes encoding angiogenic growth factors which are secreted by hypoxic cells and promote endothelial cell growth [25]. Given the apparent association of HIF-1*α* expression with IDH1 mutation, we asked whether such mutation confers angiogenic properties on the chondrosarcoma JJ012 cells. Interestingly, functional analysis of the transcriptome in JJ012 IDH1^mut^ KO cells identified angiogenesis as one of the most prominently regulated programs (adjusted *p* = 1.02*E* − 06) in chondrosarcoma. Upon further examination of angiogenic markers by IHC, we observed that CD31 and VEGFA expression in JJ012 IDH1^mut^ KO cell-derived tumors was markedly reduced when compared to parental cell-derived tumors (Figures 3(a) and 3(b)). These findings suggest a functional association of IDH1 mutation with the HIF-1*α*-driven angiogenic pathway. To further assess this functional link, we conducted vascular-endothelial tube formation assays using HUVECs incubated with the secretome of JJ012 parental or IDH1^mut^ KO cells. We found that culturing with the DH1^mut^ KO secretome significantly inhibited HUVECs ability to form vascular tubes and reduced capillary tube length by over 20% (*p* < 0.05) (Figure 3(c)). These findings indicate that IDH1-mutant JJ012 chondrosarcoma cells produce a secretome highly capable of stimulating angiogenesis and promoting tumor growth.

### 3.4. HIF-1*α* Contributes to Tumorigenicity of IDH1 Mutation in JJ012 Cells

**Figure 4:**
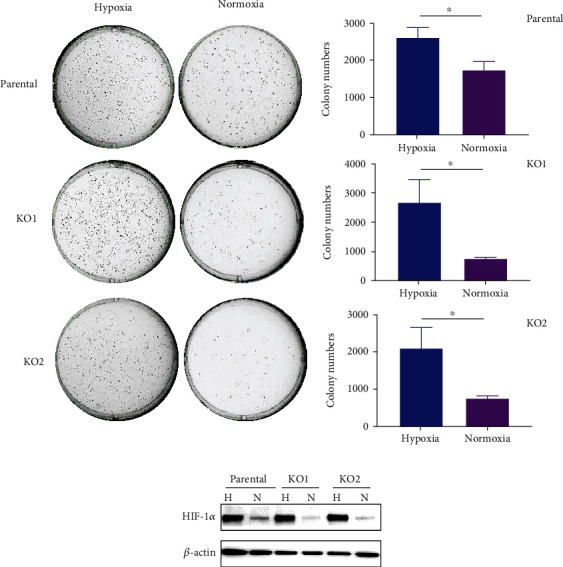
HIF-1*α* contributes to the tumorigenic function of IDH1 mutation in JJ012 cells. (a) Colony formation assay with JJ012 parental and IDH1^mut^ KO cells under hypoxic (1% O_2_) and normoxic (21% O_2_) conditions. 5 × 10^3^ cells per well were seeded in 6-well plates and incubated for 10–14 days. Graphs compare effects of hypoxia and normoxia on the number of colonies in the parental, KO1, and KO2 groups. Data indicate mean ± SEM of triplicate cultures and are representative of 3 independent experiments. ∗*p* < 0.05. (b) Immunoblot shows HIF-1*α* levels in JJ012 parental and IDH1^mut^ KO cells under normoxia (N) and hypoxia (H) conditions.

HIF-1*α* regulates gene expression in critical pathways that drive tumorigenesis [25]. Thus, we endeavored to determine whether HIF-1*α* contributes to the oncogenic properties of IDH mutation in the JJ012 chondrosarcoma cells. One of the defining criteria of tumorigenicity is anchorage-independent cell growth [26]. In our previous study, we demonstrated that loss of IDH1^mut^ attenuated the tumorigenic potential of chondrosarcoma cells. In particular, depletion of IDH1^mut^ led to a marked reduction in JJ012 capacity for anchorage-independent growth in soft agar [22]. To verify whether HIF-1*α* contributes to the observed promotion of colony formation by IDH mutation, we performed this assay under conditions of hypoxia (1% O_2_) and normoxia (21% O_2_) using JJ012 parental and IDH1^mut^ KO cells. We observed that hypoxia caused a dramatic increase in colony numbers in the IDH1^mut^ KO groups (over 60%; *p* < 0.05), compared with those grown under normoxic conditions (Figure 4(a)). A similar pattern was also seen in the parental cells (Figure 4(a)). Notably, growth in hypoxia appeared to abolish the previously reported difference in colony formation between the parental and IDH1^mut^ KO JJ012 cells performed in a normoxic atmosphere (22). HIF-1*α* levels under both conditions were analyzed by immunoblotting. As expected, incubation with 1% O_2_ stimulated HIF-1*α* expression to comparable levels in all three cell groups (Figure 4(b)). Together, these results suggest that IDH1 mutation contributes to JJ012 cells oncogenic functions, at least in part through HIF-1*α* activation.

## 4. Discussion

Tumor recurrence and metastasis are major challenges in the treatment of chondrosarcomas. Metastatic chondrosarcoma has a dismal prognosis due to lack of effective systemic therapies. IDH1/2 mutations have been frequently found in chondrosarcoma and have become an attractive target for IDH-mutant advanced chondrosarcomas. By means of drug inhibition and CRISPR/Cas9 knockout of IDH1^mut^, our previous studies have implicated IDH mutation in chondrosarcoma tumorigenicity, *in vitro* and *in vivo* [22, 24]. However, the underlying mechanism remains largely unknown. It has been proposed that D-2HG at high levels acts as an oncometabolite and exerts some potential pro-tumorigenic effects by competitively inhibiting *α*-KG-dependent enzymes such as PHDs which regulate HIF-1*α* stability. Studies have shown IDH mutations compromise the activity of PhD and stabilize HIF-1*α* in glioma cells under normoxic conditions, leading to inappropriate activation of its target genes [18, 19]. Moreover, HIF-1*α* and its target genes such as Glut1, VEGF, and PGK1 are also upregulated in the brains of IDH1 R132H knock-in mice [27]. Consistently, in our study, RNA-Seq data analysis of chondrosarcoma JJ012 cells revealed downregulation of several HIF-1*α* target genes upon loss of IDH1^mut^. This is correlated with reduced HIF-1*α* levels in these IDH1^mut^ KO cells and tumors compared with their parental controls. These findings are suggestive of a similar association between IDH mutation and HIF-1*α* induction in chondrosarcoma cells.

HIF1*α* is a key component of HIF1, a transcription factor that senses low cellular oxygen levels and regulates the expression of genes implicated in glucose metabolism, angiogenesis, and other signaling pathways that are critical to tumor growth. Increased expression of HIF-1*α* is closely associated with tumor progression in various cancers [28, 29]. Interestingly, two studies evaluated the expression of HIF-1*α* in cartilage tumors and suggested that HIF-1*α* expression was significantly correlated with shorter disease-free survival in chondrosarcoma [30, 31]. To determine whether HIF-1*α* is a contributor in IDH mutation-driven tumorigenesis of chondrosarcoma, we examined the capacity of JJ012 parental and IDH1^mut^ KO cells for anchorage-independent growth under normoxia and hypoxia conditions in a soft-agar colony formation assay. We observed that IDH1^mut^ knockout cells formed less colonies than parental cells under normoxia condition, but exogenous induction of HIF-1*α* significantly boosted the colony-forming capacity of these cells to a degree that is comparable with that of the parental cells. This suggests that activation of HIF-1*α* signaling is involved in the tumorigenic activity of IDH1^mut^*in vitro*. Of note, our previous study has shown that loss of IDH1^mut^ led to a marked attenuation of chondrosarcoma tumor formation and D-2HG production in a xenograft model [22]. Since anchorage-independent growth is tightly correlated with tumorigenic potential *in vivo*, it is conceivable that the attenuated HIF-1*α* signaling caused by loss of IDH1^mut^ might contribute to the observed inhibition of chondrosarcoma formation in the xenograft model.

Angiogenesis is a key contributor to tumor progression and metastasis. HIF-1*α* and VEGF are known to play crucial roles in the tumor angiogenic process [12, 25, 32]. It is established that VEGF expression is mediated by HIF-1*α* during hypoxia. The VEGF gene contains a number of HIF-1*α*-binding sites at its regulatory region, and HIF-1*α* is able to activate the VEGF promoter [33, 34]. HIF-1*α*-induced VEGF expression is implicated in the angiogenic switch in chondrosarcoma [32]. Interestingly, studies have shown that IDH mutation is associated with elevated levels of HIF-1*α* and VEGF levels in IDH-mutant gliomas [18, 35]. Consistently, we found the expression of angiogenic markers, VEGFA and CD31, was significantly reduced in the IDH1^mut^ KO cell-derived tumors, suggesting that IDH1 mutation is associated with the angiogenic potential of chondrosarcoma cells. This association was solidified by *in vitro* vascular tube formation assay, which shows that the secretome of IDH1^mut^ KO cells substantially reduced HUVECs' ability to from primitive vascular tubes in comparison with the secretome of parental cells. To be noted, our previous study also showed an association between IDH mutation and aberrant activation of integrin signaling in chondrosarcoma cells [22]. A few integrins have been implicated in blood vessel formation and regulation of cell growth, survival, and migration during tumor angiogenesis and metastasis [36, 37]. Interestingly, although VEGF expression is induced by HIF-1*α* during hypoxia [38], its expression can also be modulated by tumor integrins, resulting in efficient tumor angiogenesis under normoxic conditions [39]. Therefore, integrin and HIF-1*α* signaling might intertwine with each other by shared mediators such as angiogenic factors in the tumor microenvironment, and thus both pathways contribute to the process of angiogenesis in IDH-mutant chondrosarcomas [12, 36]. In any case, the established association in this study renders antiangiogenic molecules appealing candidates for combinatorial regimens with IDH1^mut^ inhibitors for advanced chondrosarcomas. The use of angiogenesis inhibitors has been used as an adjunct to other forms of therapy for preventing development of malignant neoplasms [40]. Preclinical studies have shown the benefits of targeting angiogenesis in chondrosarcomas [41]. Given the modest results with IDH1^mut^ inhibitors in chondrosarcoma [42], future efforts to improve the efficacy of these compounds might benefit from emphasis on biology-driven therapeutic strategies to improve response rates in IDH-mutated chondrosarcomas.

The present study was limited by the use of only one conventional chondrosarcoma cell line. In fact, due to the rareness of this malignancy, very few chondrosarcoma cell lines are available worldwide. Thus, more chondrosarcoma cell lines, or patients' primary tumor cells, will be essential to further investigate and strengthen the concept explored in this study. Interestingly, we did not find a similar association between IDH mutation and HIF-1*α* in an IDH1-mutant fibrosarcoma cell line, HT1080, which was originally reported as a fibrosarcoma of bone, but is now considered to represent a dedifferentiated chondrosarcoma. We believe that the discrepancy is due to pathogenesis heterogeneity between the chondrosarcoma cell lines as conventional and dedifferentiated chondrosarcomas were shown to exhibit distinct biological behaviors and clinical characteristics. As described previously, differential biology was also observed within gliomas and leukemias with regards to the effects of IDH mutation on PHDs and HIF-1*α* activity [18–21]. Thus, much work remains to better understand the biology to fully clarify these discrepancies and identify the appropriate patient populations for specific targeted therapies. Nonetheless, herein, we identified a strong correlation between HIF-1*α* activation and IDH1 mutation status in chondrosarcoma cells. Furthermore, this study unravels one aspect of chondrosarcoma pathophysiology and provides insightful therapeutic possibilities such as combinatorial regimens of antiangiogenic agents with IDH1^mut^ inhibitors for patients with advanced IDH mutated chondrosarcoma.

## Data Availability

The experimental data used to support the findings of this study are included within the article.
